# A cold scare: Formation of cold reactive anti-A1 coinciding with gross hemolysis

**DOI:** 10.1016/j.plabm.2018.e00100

**Published:** 2018-05-07

**Authors:** Jeffrey Petersen, Darshana Jhala

**Affiliations:** a Department of Pathology and Laboratory Medicine, Michael J. Crescenz Veteran Affairs, Medical Center, 3900 Woodland Avenue, Philadelphia, PA 19104, United States; b University of Pennsylvania Perelman School of Medicine, United States

**Keywords:** Immunohematology, Transfusion medicine, Blood transfusion, Hemolysis/immunology, Transfusion reactions, Laboratory medicine

## Abstract

Anti-A_1_ antibodies can be found as a usually clinically insignificant naturally occurring cold IgM antibody in A-subgroup patients. It is known from multiple prior case reports that warm-reactive anti-A_1_ that reacts at 37 °C can be clinically significant, and it has been previously reported that it could form after alloimmunization with donor A_1_ red blood cell (RBC) transfusion. In addition, the development of anti-A_1_, often as an autoantibody, have been described in the setting of various malignancies, perhaps due to expressed subtle alterations of the ABO antigens provoking an immune response.

Here, we report a rare case of a cold-reactive anti-A_1_ alloantibody (after multiple transfusions with group A_1_ RBC units) in a 76 year old male patient (A_2_) with history of myelodysplastic syndrome and metastatic carcinoma who presented with hemolytic anemia and dark urine. The patient had previously typed as blood type A without reverse typing reaction for anti-A_1_; as a result, the patient had been transfused with group A_1_ RBCs. Four days prior to discovery of the ABO discrepancy, the patient had a febrile transfusion reaction associated with his A_1_ RBC transfusion. On admission, his immunohematology workup demonstrated an alloantibody to anti-A_1_ that coincidentally appeared during a new onset of hemolytic anemia. Case reports of patients with hemolytic anemia with a newly developed anti-A_1_ alloantibody are sparse in the literature, and this case is particularly interesting as the cold reactive anti-A_1_ (without demonstrable wide thermal amplitude) appeared to form after alloimmunization and in the setting of an underlying malignancy.

## Highlights

• Anti-A_1_ alloantibodies are usually clinically insignificant naturally occurring cold IgM antibodies that can occur in some A-subgroup patients.
• Anti-A_1_ have been reported to be clinically significant if the antibody is reactive at 37 °C or body temperature on immunohematology testing.
• Rare case reports exist of anti-A_1_ forming either after alloimmunization with exposure to A_1_ antigen or forming in the context of a paraneoplastic process.
• This case is a report of an alloantibody to anti-A_1_ forming in the context of both alloimmunization and in the context of an underlying metastatic carcinoma.

## 1 Introduction

ABO antigens are present on red blood cells (RBCs) and the majority of individuals (approximately 80%) typing as ABO group A express the A_1_ antigen on their RBCs [1], [2]. Anti-A_1_ is occasionally found as a naturally occurring alloantibody in the serum of around 1–8% of A_2_ individuals and 22–35% of A_2_B individuals [1], [2], [3], [4]. As anti-A_1_ is generally an IgM antibody that reacts best at room temperature or below, it is unusually considered to be clinically insignificant [1], [2], [3], [4], [5], [6]. The presence of anti-A_1_ in the overwhelming majority of cases does not necessarily preclude the use of A_1_ red blood cells (RBCs). However, rare clinically significant cases have been noted when the antibody demonstrated reactivity at 37 °C; in some of these cases, acute or delayed hemolytic transfusion reactions have been documented as case reports in the literature [6], [7], [8]. Among these clinically significant cases in which the antibody had a wide thermal amplitude, rarer case reports have noted apparent development after alloimmunization with A_1_ antigen exposure or development of an “auto-anti-A_1_” in association with metastatic carcinoma [5], [9], [10]. Anti-A_1_ antibodies with a significant autoimmune component have also been reported as arising after alloimmunization to A_1_ antigen, in the setting of a malignancy, or idiopathically in an A_1_ blood group patient shortly before death from severe intravascular hemolysis [5], [9], [10].

Here, we report a rare case of a cold-reactive anti-A_1_ antibody that formed as an alloantibody (after multiple transfusions with group A_1_ RBC units) in a 76 year old male patient (A_2_) with history of myelodysplastic syndrome who presented with autoimmune hemolytic anemia and dark urine; on discharge, the patient also was newly diagnosed with metastatic carcinoma (likely urothelial in origin) on his bone marrow biopsy. Prior to presentation with the anti-A_1_, the patient had previously typed repeatedly via tube method as blood type A without any reverse typing reaction for anti-A_1_; as a result, the patient had been transfused with group A_1_ RBCs over the course of two weeks. On admission after these transfusions, the patient was found to have developed a new cold-reactive anti-A_1_ alloantibody in the setting of gross hemolysis; this unusual presentation after apparent alloimmunization with a literature review is discussed. Case reports of patients with hemolytic anemia with a newly developed anti-A_1_ alloantibody are sparse in the literature, and this case of hemolytic anemia is particularly interesting as the cold-reactive anti-A_1_ without demonstrable wide thermal amplitude appeared to form after alloimmunization and in the setting of an underlying malignancy.

## 2 Report of a case

Reported is a 76 year old male patient with history of myelodysplastic syndrome and osteoporosis who presented initially with diffuse musculoskeletal pain, a 20–30 lb. weight loss over the past year, and weakness for the past two months that worsened 1 week prior to presentation. His initial laboratory values demonstrated a pancytopenia with hemoglobin of 5.6 g/dL that was clinically felt to likely represent a chronic progression of his myelodysplastic syndrome (MDS); given his anemia, the transfusion of two units of group A red blood cells (RBCs) was ordered and the appropriate pre-transfusion testing was performed (see Table 1). Notably, at the time of initial presentation for transfusion, the pre-transfusion blood bank workup did not demonstrate an ABO discrepancy or the presence of an anti-A1 on reverse typing. This is consistent with the patient's long history of blood bank testing extending over 5 years prior to this day in which the patient had repeatedly tested A+ with a negative antibody screen and without an ABO discrepancy. Despite the long documented ABO typing history, the patient had not been previously transfused at the hospital. The patient received the two units of A_1_+ RBCs without complication or transfusion reaction and had an appropriate increase of his hemoglobin to 7.6 g/dL the following day. After the transfusion, the patient reported feeling somewhat better and without focal pain.

**Table 1:** with initial pre-transfusion laboratory values.

Initial pre-transfusion laboratory values				
	Value at initial hospitalization	Value at 2nd Visit 6 days after discharge from first hospitalization	Value at 3rd Visit 3 days after discharge from 2nd visit	Reference range
ABO Rh	A+	A+	ABO discrepancy	
Antibody Screen	Negative	Negative	Negative	Negative
Electronic Crossmatch with A+ units selected for transfusion	Compatible	Compatible	Serologically compatible Group O red blood cell units provided	Compatible
No reaction to A_1_ on reverse typing on ABO blood group testing at either visit #1 or 2.			Reaction to A_1_	

On his third hospital day, the patient developed febrile neutropenia (spike in temperature to 101.9 F with an absolute neutrophil count of 0.5 × 10^3^/mm^3^) without a clear source though at the time, the patient's posterior neck rash (for folliculitis) versus a urinary tract origin given his urinary incontinence were considered. The patient was treated appropriately with antibiotics and, as he had clinically improved by his fourth hospital day with negative blood culture and contaminated urine culture, he was discharged on a seven day course of PO levofloxacin and with hemoglobin at discharge of 7.9 g/dL.

Six days after discharge, the patient returned to the hospital for his 2nd hospitalization presenting with decreased energy and appetite. On presentation, the patient was anemic with a hemoglobin value of 7.2 g/dL and neutropenic with an absolute neutrophil count of 0.6 × 10^3^/mm^3^. As the patient had symptomatic anemia, the decision was made to transfuse the patient with two group A_1_+ RBC units. Prior to the transfusion of the second unit, the patient's pre-transfusion vitals were stable as listed on Table 2; of note, the patient's temperature was 99.2 F. After the transfusion, the patient developed a fever with a temperature of 102.5 F, but with no other symptoms and post-transfusion vital signs as per Table 2. The patient was treated with acetaminophen, ibuprofen, and intravenous fluids with resolution of the fever. A transfusion reaction workup and additional laboratory studies for hemolysis was sent (see Table 2). Of note, the direct antiglobulin test (DAT) was positive (1+) for complement on both the pre- and post- transfusion specimens. Based on the clinical history and laboratory testing results, the reaction was felt to likely be a febrile non-hemolytic transfusion reaction. As the patient had improved clinically with a post-transfusion hemoglobin value of 8.6 g/dL, the patient was discharged with planned follow-up with hematology/oncology as an outpatient.

**Table 2:** with Transfusion Reaction vitals and Transfusion Reaction workup testing results. Abbreviations: DAT = direct antiglobulin test, Hgb = hemoglobin.

Transfusion reaction vitals at 2nd hospitalization		
Vital sign	Pre-transfusion	Post-transfusion
Temperature	99.2 F	102.5 F
Pulse	65 beats/minute	90 beats/minute
Blood pressure	129/65 mmHg	141/79 mmHg
Respiratory rate	18	18
Transfusion reaction workup (below)		
Test	Result	Reference or expected value
Clerical check	Passed	Passed
Repeat ABO Rh	A+	A+
Both Units' Blood Type	A+	A+
Repeat Antibody Screen	Negative	Negative
Visual Hemolysis of pre-transfusion specimen	Negative	Negative
Visual Hemolysis of post-transfusion specimen	Negative	Negative
Pre-transfusion DAT	1+ for complement	Negative
Post-transfusion DAT	1+ for complement	Negative
Haptoglobin	134 mg/dL	36–395 mg/dL
Bilirubin, total	3.1 mg/dL	0.4–2.0 mg/dL
Bilirubin, direct	0.7 mg/dL	0.1–0.5 mg/dL
Post-transfusion Hgb	8.7 g/dL	
Urine color	Amber	Yellow
Urine blood	Negative	Negative

**Fig. 1:**
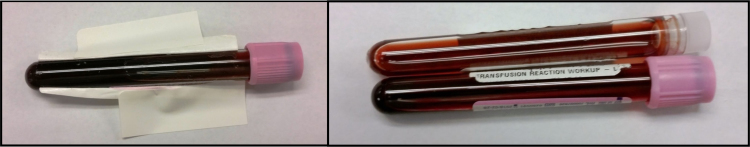
Photograph of grossly hemolyzed blood specimen.

Three days after discharge from the 2nd hospitalization, the patient returned with dark bloody urine since one day prior to admission for the 3rd hospitalization as well as increased lethargy and weakness. The patient's presenting hemoglobin had declined to 6.3 g/dL on admission. Given the patient's anemia, the clinical decision was made to transfuse the patient and admit the patient to the intensive care unit. The pre-transfusion specimens received at the blood bank appeared grossly hemolyzed (1+) and workup revealed an ABO discrepancy due to reactivity to A_1_ cells on reverse ABO typing (tube method) with negative antibody screen (tube method with low ionic strength saline - LISS). Due to the ABO discrepancy and the unusual case presentation, patient blood samples were sent to the American Red Cross (ARC) for further investigation. Anti-A_1_ lectin (Dolichos biflores) demonstrated a mixed field on in-house testing, likely due to recent transfusions of A_1_ RBC units. Of note, as part of the in-house immunohematology workup, four units of group A RBCs were also serologically crossmatched (by immediate spin, 37 C, and anti-human globulin – AHG - phase) via tube method and LISS (at 37 C and AHG phase) and two out of the four were incompatible. One A+ unit was incompatible 3+ at immediate spin, 2+ at 37C, and 1+ at the AHG phase. The other incompatible A+ unit was incompatible 3+ at immediate spin, not reactive at 37C, and questionable (+/-) incompatibility at AHG phase. In contrast, all of the selected O+ units were compatible on serologic crossmatch using the same methodology. After discussion with the patient's treating clinicians, two serologically crossmatch compatible (by immediate spin, 37C, and AHG phase) group O RBCs was provided for transfusion. While vital signs were stable prior to and immediately after transfusion, the patient developed symptoms three hours after the end of his second transfusion; these symptoms included fever to 104.2 F with a transient pulse oximeter reading of 91% on room air from 98%. These symptoms resolved with acetaminophen administration and provision of 2 l oxygen by nasal cannula. As part of the transfusion reaction workup, a post-transfusion blood specimen (see Fig. 1) was drawn and this specimen was also sent to the ARC for immunohematology testing after the initial on-site transfusion reaction workup.

The immunohematology findings from ARC demonstrated that the patient was A_2_D positive (not group A_1_) with an alloantibody (anti-A_1_) identified on the serum antibody identification (see Table 3). The reactivity of the anti-A_1_ was removed by the prewarm technique (37 °C settle and IgG-antiglobulin testing). The direct antiglobulin test demonstrated reactivity as in Table 3 with a post-transfusion pan-reactive acid eluate with no specificity. No other alloantibodies were identified on testing.

**Table 3:** Additional laboratory values for 3rd hospitalization.

**Direct antiglobulin test results at american red cross**		
	Pre-transfusion	Post-transfusion
Polyspecific anti-human globulin	+^m^	1+
Monospecific anti-IgG	Negative	1+
Monospecific anti-C3d	+^m^	+^m^
Saline Control	Negative	Negative
**Serum Antibody Identification and Eluate Results at American Red Cross**		
**Anti-A**_**1**_	IS, RT, 37 °C-ALB, ALB-IgG-AGT (reactivity removed by prewarm technique −37 °C settle and IgG-AGT)	
**Eluate**	Panreactive with all group O and group A reagent red cells tested at IgG-AGT phase.	

## 3 Clinical course

During the patient's treatment in the intensive care unit, the patient was given group O RBCs with an appropriate increase in hemoglobin. Given the unusual presentation of a cold reactive anti-A_1_ with gross hemolysis, the provision of serologically crossmatch-compatible group O RBCs was felt to be the most prudent transfusion choice.

However, due to ongoing hemolytic anemia, after the initial appropriate increase in hemoglobin to transfusion, the patient's hemoglobin would subsequently drop during the hospitalization despite repeated transfusions with compatible group O RBCs. To treat the patient's underlying hemolytic anemia, the patient also received methylprednisolone starting on day 2 of hospitalization and later rituximab starting on day 5 of admission. The patient became independent from transfusion by day 5 of hospitalization with stabilization of the patient's hemoglobin to 9.7 g/dL at discharge nine days after admission. Throughout his 3rd hospital course, the patient received six units of group O RBCs (2 on admission to hospital day 1, 2 on hospital day 3, and 2 on hospital day 4).

While the patient's need for RBC transfusion and anemia improved during the hospitalization, the patient was diagnosed by left posterior iliac crest bone marrow biopsy with metastatic carcinoma favor urothelial based on immunohistochemical studies. Due to the patient's prognosis from his metastatic carcinoma and the patient's wishes on discussion with the clinical team, the patient entered hospice at home.

## 4 Discussion

As a number of tissues such as urothelium can express ABO-related antigens and corresponding carcinomas can express these antigens (perhaps with subtle modification), it had previously been speculated that this antigen expression could provoke an immune response in the host [9], [11], [12]. On the other hand, in a patient who is blood group A_2_, an immune response provoked by alloimmunization has been reported rarely, with one case series of two patients demonstrating an increase in titer and thermal amplitude of the normally weak naturally-occurring anti-A_1_ [4]. A general unifying theme of these reported clinically significant anti-A_1_ antibodies has been that the antibody demonstrated a wide thermal amplitude [1], [2], [3], [4], [5], [6], [7], [8].

In our described case report, the patient was transfused with A_1_ red blood cell units that could have led to alloimmunization, but also had an underlying likely urothelial carcinoma. Prior case reports have demonstrated patients developing anti-A_1_ with associated hemolysis after either alloimmunization by transfusion or after diagnosis of an underlying malignancy that could have expressed an altered A antigen.

## 5 Conclusion

While hemolysis from anti-A_1_ tends to be the exception rather than the rule, knowledge about these rare cases may impact the care of patients with ABO discrepancies who may be developing an anti-A_1_.

## Conflicts of interest

None.

## Funding

This research did not receive any specific grant from funding agencies in the public, commercial, or not-for-profit sectors.

## References

[1] M.K. Fung, editor. AABB Technical Manual. 18th edition. Bethesda, MD, USA: AABB. Chapter 12. ABO, H, and Lewis Blood Groups and Structurally Related Antigens.

[2] Reid M.E., Lomas-Francis C., Olsson M.L. (2012). The Blood Group Antigen FactsBook.

[3] Domen R.E., Calero A., Keehn W.H. (1988). Acute hemolytic transfusion reaction due to anti-A_1_. Lab. Med..

[4] Jakobowicz R., Simmons R.T., Carew J.P. (1961). Group A blood incompatibility due to the development of apparent anti-A_1_ antibodies in a patient of subgroup A_2_. Vox Sang..

[5] Contreras M., Hazlehurst G.R., Armitage S.E. (1983). Development of ‘auto-anti-A_1_ antibodies' following alloimmunization in an A_2_ recipient. Br. J. Haematol..

[6] Northoff H., Wӧlpl A., Sugg U. (1986). An unusual sample of irregular anti-A_1_, probably causing an early delayed transfusion reaction. BLUT.

[7] Preece J., Magrin G., Webb A. (2011). A *bloody* mistake: unrecognized warm reactive anti-A1 resulting in acute hemolytic transfusion reaction. Transfusion.

[8] Lundberg W.B., McGinniss M.H. (1975). Hemolytic transfusion reaction due to anti-A_1_. Transfusion.

[9] Castella A., LaBarge B.P., Lauenstein K.J. (1983). Auto-anti-A_1_ in a patient with metastatic adenocarcinoma. Transfusion.

[10] Szymanski I.O., Roberts P.L., Rosenfield R.E. (1976). Anti-A autoantibody with severe intravascular hemolysis. N. Engl. J. Med..

[11] Kovarik S., Davidsohn L., Stejskal R. (1968). ABO antigens in cancer: detection with the mixed cell agglutination reaction. Arch. Pathol..

[12] Orntoft T.F., Wolf H., Clausen H. (1988). Blood group ABO-related antigens in fetal and normal adult bladder urothelium. Immunohistochemical study of type 2 chain structures with a panel of mouse monoclonal antibodies. Lab Investig..

